# Prospective Feasibility Study for Using Cell-Free Circulating Tumor DNA–Guided Therapy in Refractory Metastatic Solid Cancers: An Interim Analysis

**DOI:** 10.1200/PO.16.00059

**Published:** 2017-06-26

**Authors:** Seung Tae Kim, Kimberly C. Banks, Se-Hoon Lee, Kyung Kim, Joon Oh Park, Se Hoon Park, Young Suk Park, Ho Yeong Lim, Won Ki Kang, Richard B. Lanman, AmirAli Talasaz, Keunchil Park, Jeeyun Lee

**Affiliations:** **Seung Tae Kim**, **Se Hoon Park**, **Kyung Kim**, **Joon Oh Park**, **Se-Hoon Lee**, **Young Suk Park**, **Ho Yeong Lim**, **Won Ki Kang**, **Keunchil Park**, and **Jeeyun Lee**, Samsung Medical Center, Sungkyunkwan University School of Medicine, Seoul, Korea; and **Kimberly C. Banks**, **Richard B. Lanman**, and **AmirAli Talasaz**, Guardant Health, Redwood City, CA.

## Abstract

**Purpose:**

Retrospective studies have demonstrated that cell-free circulating tumor DNA (ctDNA) hotspot testing predicts matched therapy response to first- and second-line therapies in patients with advanced non–small-cell lung cancer (NSCLC). However, no prospective outcomes studies have evaluated ctDNA-guided matched therapy decision making on the basis of comprehensive plasma genomic testing including all four major classes of alterations. Here, we report the clinical utility of this approach in advanced solid tumor cancers.

**Patients and Methods:**

We conducted a multiple parallel cohort, open-label, clinical trial using ctDNA-guided matched therapy when tissue was insufficient or unobtainable for next-generation sequencing. Plasma-based digital sequencing identified point mutations in 70 genes and indels, fusions, and copy number amplifications in selected genes. Patients with prespecified targetable alterations in metastatic NSCLC, gastric cancer (GC), and other cancers were matched to several independent targeted agent trials at a tertiary academic center.

**Results:**

Somatic alterations were detected in 59 patients with GC (78%), and 25 patients (33%) had targetable alterations (*ERBB2*, n = 11; *MET*, n = 5; *FGFR2*, n = 3; *PIK3CA*, n = 6). In NSCLC, 62 patients (85%) had somatic alterations, and 34 (47%) had targetable alterations (*EGFR*, n = 29; *ALK*, n = 2; *RET*, n = 1; *ERBB2*, n = 2). After confirmation of ctDNA findings on tissue (to meet trial eligibility criteria), 10 patients with GC and 17 patients with NSCLC received molecularly matched therapy. Response rate and disease control rate were 67% and 100%, respectively, in GC and 87% and 100%, respectively, in NSCLC. Response was independent of targeted alteration variant allele fraction in NSCLC (*P* = .63).

**Conclusion:**

To our knowledge, this is the first prospective feasibility study of comprehensive ctDNA-guided treatment in advanced GC and lung cancers. Response rates in this interim analysis are similar to those in tissue-based targeted therapy studies.

## INTRODUCTION

The National Comprehensive Cancer Network guidelines recommend genotyping in seven solid tumor cancers (non–small-cell lung cancer [NSCLC], breast, gastric, esophageal, colorectal, melanoma, and GI stromal tumors) for 11 genomic targets (GTs) to inform targeted therapy selection.^1-6^ However, biopsy specimens can be inadequate for comprehensive profiling in 25% to 50% of patients,^7-10^ leading to incomplete genotyping or repeat invasive biopsy to obtain more tissue. Repeat biopsy is also recommended at progression in patients with breast cancer and NSCLC to capture targetable genomic changes such as *ERBB2* (human epidermal growth factor receptor 2 [HER2]) copy number amplification (CNA) or *EGFR* and *ALK* resistance mutations, respectively.^4,5,11^

Comprehensive ctDNA testing covering point mutations, insertions/deletions (indels), fusions, and CNA may obviate the need for repeat invasive biopsies for genotyping when tissue is of insufficient quantity or unobtainable at initial diagnosis or at progression.^12,13^ In general, next-generation sequencing (NGS) seems to detect more actionable variants in target genes than non-NGS methods (hotspot testing) such as polymerase chain reaction (PCR), immunohistochemistry (IHC), or fluorescence in situ hybridization.^14-18^ Beyond the benefits of invasive biopsy avoidance and higher sensitivity compared with non-NGS methods, comprehensive ctDNA NGS may provide a global summary of multiple lesions, whereas tissue genotyping of small biopsies may fail to capture intra- and intertumor heterogeneity.^19-21^

Retrospective studies in NSCLC using ctDNA genotyping for *EGFR* mutations in the first-line (*EGFR*^L858R/exon19del^)^22^ and second-line (*EGFR*^T790M^)^23,24^ settings have produced response rates similar to studies of therapies directed by tissue-based genotyping. A small study of ctDNA-identified *ERBB2* (HER2) CNA in metastatic breast cancer found an 86% response rate to anti-HER2 treatment.^25^ No prospective outcomes studies have evaluated comprehensive ctDNA NGS testing for all four types of genomic alterations to guide matched therapy decision making in patients with advanced solid cancers.

Previously, we conducted a prospective external validation study (Next-Generation Personalized Therapy With Plasma DNA Genomics Trial [NEXT] -1) of a 54-gene ctDNA NGS test (Guardant360; Guardant Health, Redwood City, CA), finding 86% concordance between pretreated matched plasma and tissue samples in multiple advanced solid tumor cancer types.^26^ Now expanded to 70 genes covering all four major types of targetable genomic alterations,^27^ we hypothesized that this comprehensive ctDNA digital sequencing test could effectively guide targeted therapy in patients with metastatic NSCLC, gastric cancer (GC), and other cancers.

## PATIENTS AND METHODS

### Study Design and Treatment

**Fig A1.**
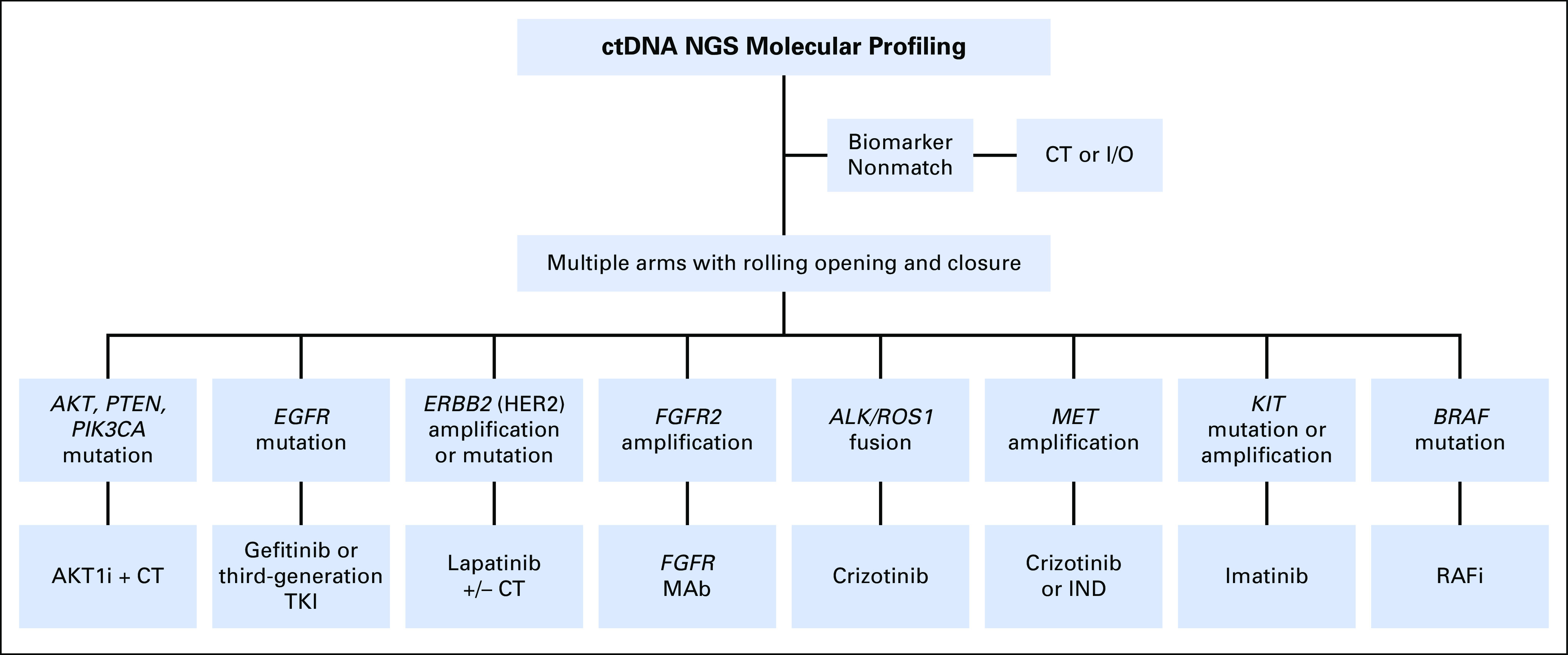
NEXT-2 clinical trial design: matched therapy protocols aligned to the institutional review board–approved NEXT-2 master protocol. AKT1i, AKT1 inhibitor; CT, chemotherapy; ctDNA, circulating tumor DNA; IND, investigational new drug; I/O, immunotherapy; MAb, monoclonal antibody; NGS, next-generation sequencing; RAFi, RAF inhibitor; TKI, tyrosine kinase inhibitor.

The NEXT-2 trial in refractory solid tumors (ClinicalTrials.gov identifier: NCT02140463) consists of several matched therapy protocols (phases II to IV; Appendix Fig A1) aligned to the institutional review board–approved NEXT-2 master protocol at a single center (Samsung Medical Center, Sungkyunkwan University School of Medicine, Seoul) in the Republic of Korea. Prespecified GTs included *AKT1*, *PTEN*, *PIK3CA*, and *BRAF* mutations; *EGFR*, *KIT*, and *ERBB2* (HER2) mutations or CNA; *FGFR2* CNA; and fusions in *ROS1*, *ALK*, or *NTRK1* (Appendix Fig A1). The study was conducted in accordance with the current ethical principles outlined in the Declaration of Helsinki and Good Clinical Practice guidelines.

### Patients

Eligible patients were older than age 20 years with histologically confirmed metastatic cancer, who had sufficient tumor tissue to test cancer-specific biomarkers but not to undergo comprehensive genomic profiling (NGS). Cancer-specific biomarker testing included HER2 IHC in GC, *EGFR* mutations by hotspot sequencing and ALK IHC in NSCLC, and *BRAF*^V600E^ digital PCR in melanoma. Patients had radiologically evaluable disease, adequate organ function, life expectancy ≥ 3 months from proposed first dose date, and Eastern Cooperative Oncology Group performance status (ECOG PS) of 0 to 3. Patients with double primary cancers were excluded (except for any cancer in remission for > 5 years, in situ cervical or basal cell cancer, or any resected in situ cancers).

### End Points and Assessments

The study primary and secondary end points were progression-free survival and objective response rate (RR), respectively. This prespecified interim analysis is limited to objective response for patients receiving ctDNA-directed matched therapies. RR and disease control rate (DCR = RR + stable disease) were centrally adjudicated in accordance with Response Evaluation Criteria in Solid Tumors (RECIST) version 1.1.^28^

### Statistical Analysis

Descriptive statistics were calculated for demographics, ctDNA alteration detection rate, and substudy matching. CIs for proportions were reported using Wilson’s score interval with continuity correction. Associations between RECIST 1.1 treatment response and targeted alteration variant allele fraction (VAF), ECOG PS, and line of therapy were assessed using linear regression, *t* test, and analysis of variance, respectively.

### Comprehensive Genomic Testing in Plasma

**Fig A2.**
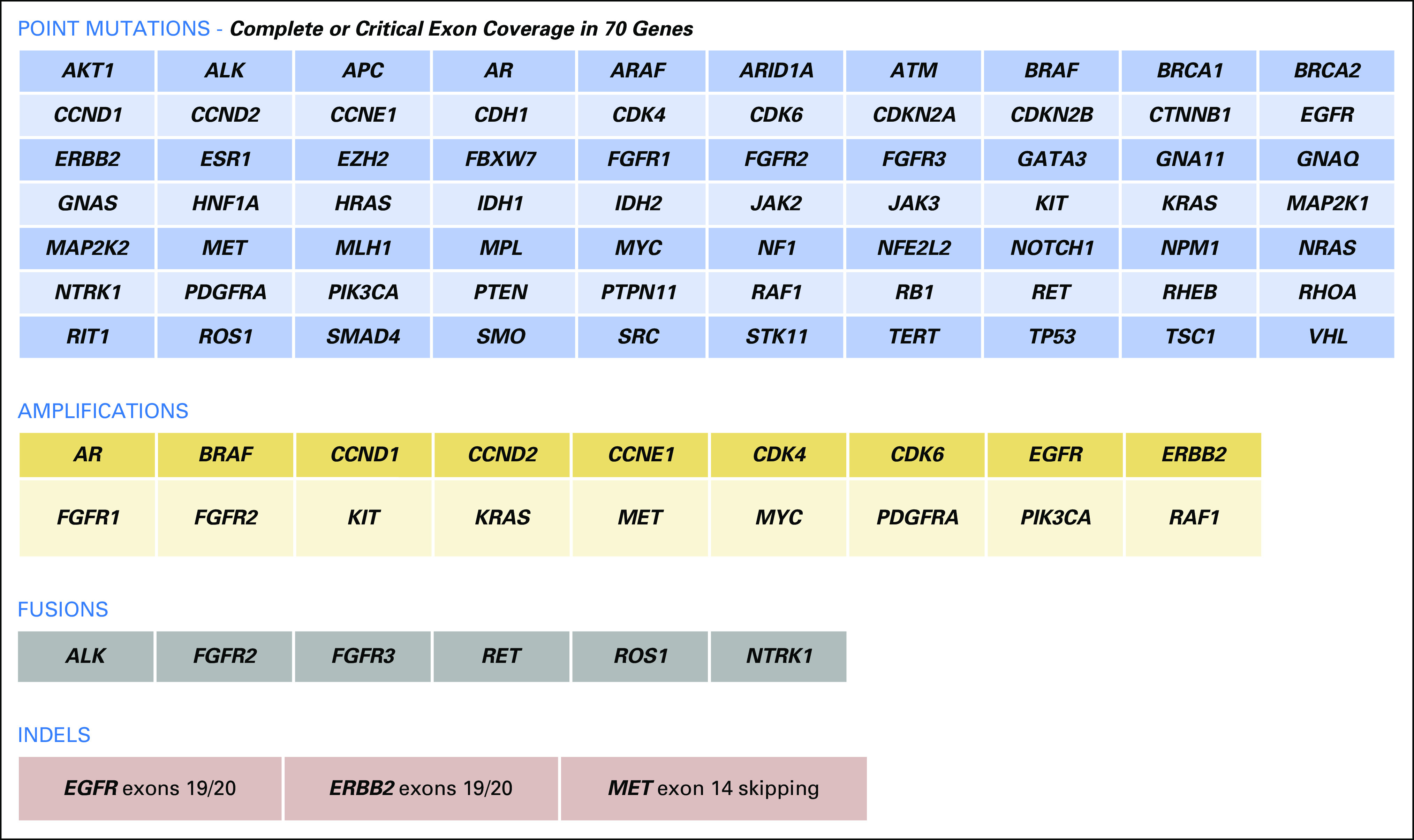
Genes covered by Guardant360 70-gene panel.

Cell-free DNA (cfDNA) was extracted from whole blood collected in 10-mL Streck tubes. Samples were shipped to a Clinical Laboratory Improvement Act–certified, College of American Pathologists–accredited laboratory (Guardant Health). After double ultracentrifugation, 5 to 30 ng of cfDNA was isolated for digital sequencing as previously described.^12,26,29^ All exons in 30 genes and critical exons (those known to harbor somatic mutations) of 40 genes were completely sequenced. Sequencing data were analyzed using a custom bioinformatics pipeline to identify single nucleotide variants (SNVs) in 70 genes (150-kb panel footprint), CNAs in 18 genes, indels in three genes (*EGFR* and *ERBB2* exons 19 and 20; *MET* exon 14), and *ALK*, *RET*, *ROS1*, *NTRK1*, *FGFR2*, and *FGFR3* fusions (Appendix Fig A2). Targetable ctDNA-detected GTs were confirmed via tissue testing.

All cfDNA fragments, both leukocyte and tumor derived, were simultaneously sequenced. The VAF was calculated as the proportion of cfDNA harboring the variant in a background of wild-type cfDNA. The analytic sensitivity reaches detection of one to two mutant fragments in a 10-mL blood sample (0.1% limit of detection) with analytic specificity > 99.9999%.^12^ CNAs were reported as the absolute gene copy number in plasma. Because most cfDNA is leukocyte derived, the gene copy number is generally 2.0. Tumor-derived DNA shed into the bloodstream increases this value but, as a result of the relative proportions of tumor-derived versus leukocyte-derived cfDNA, is typically a minor contributor. Gene copy number in plasma is thus a function of both copy number in tissue and the degree to which tumor DNA is shed into circulation. Plasma copy number of 2.5 to 4.0 is reported as ++ amplification and copy number > 4.0 as +++ amplification, representing the 50th to 90th and > 90th percentiles, respectively, of all CNA calls in the Guardant360 database.

## RESULTS

### Patient Enrollment and Demographics

**Fig A3.**
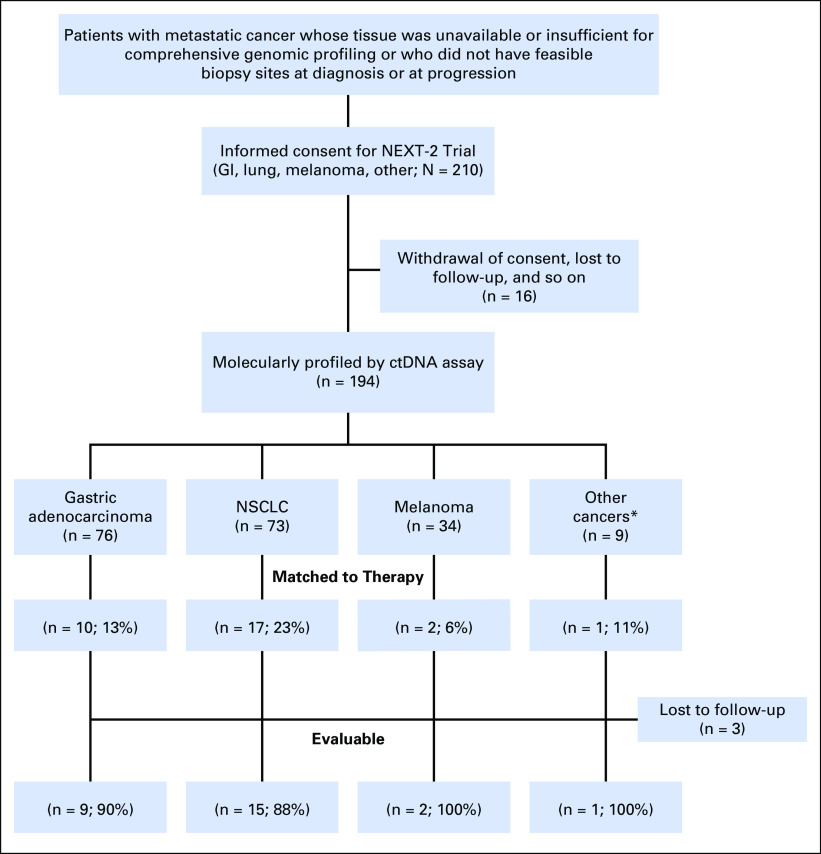
CONSORT diagram. (*) Other cancers include sarcoma, hepatocellular carcinoma, colorectal cancer, neuroendocrine tumors, skin cancers, and others. ctDNA, circulating tumor DNA; NEXT-2, Next-Generation Personalized Therapy With Plasma DNA Genomics Trial 2; NSCLC, non–small-cell lung cancer.

From August 2014 to February 2016, informed consent was obtained from 210 consecutive patients with metastatic cancer whose tissue was available for cancer-specific biomarker testing, but insufficient for NGS, at initial diagnosis or at progression. Sixteen patients were lost to follow-up or withdrew consent, leaving 194 patients molecularly profiled by ctDNA NGS (Appendix Fig A3).

Median age was 60 years (range, 28 to 78 years) for NSCLC and 57 years (range, 23 to 82 years) for GC, melanoma, and other cancers; 43%, 58%, 56%, and 89% of patients with these cancers were male, respectively (Table 1). All patients were from Korea, and the majority (85%) had an ECOG PS of 0 or 1. Newly diagnosed (first-line) patients composed 29% of patients with NSCLC, 37% of those with GC, 68% of those with melanoma, and 11% of those with other cancers; the remainder of patients were tested in the setting of second-line or greater therapy.

**Table 1.**
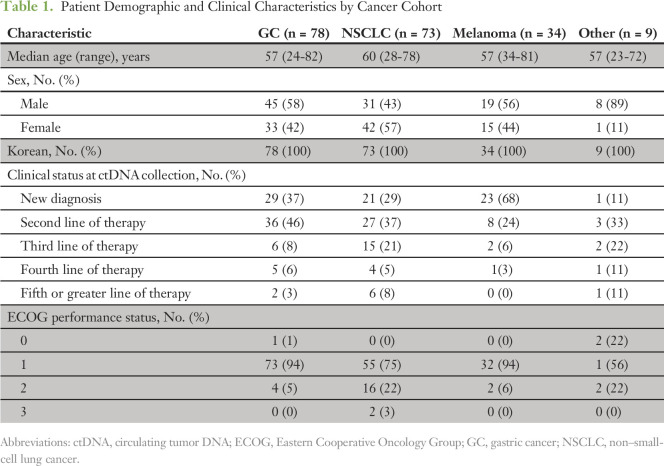
Patient Demographic and Clinical Characteristics by Cancer Cohort

### Targetable Alterations and Therapy Matching

**Fig 1.**
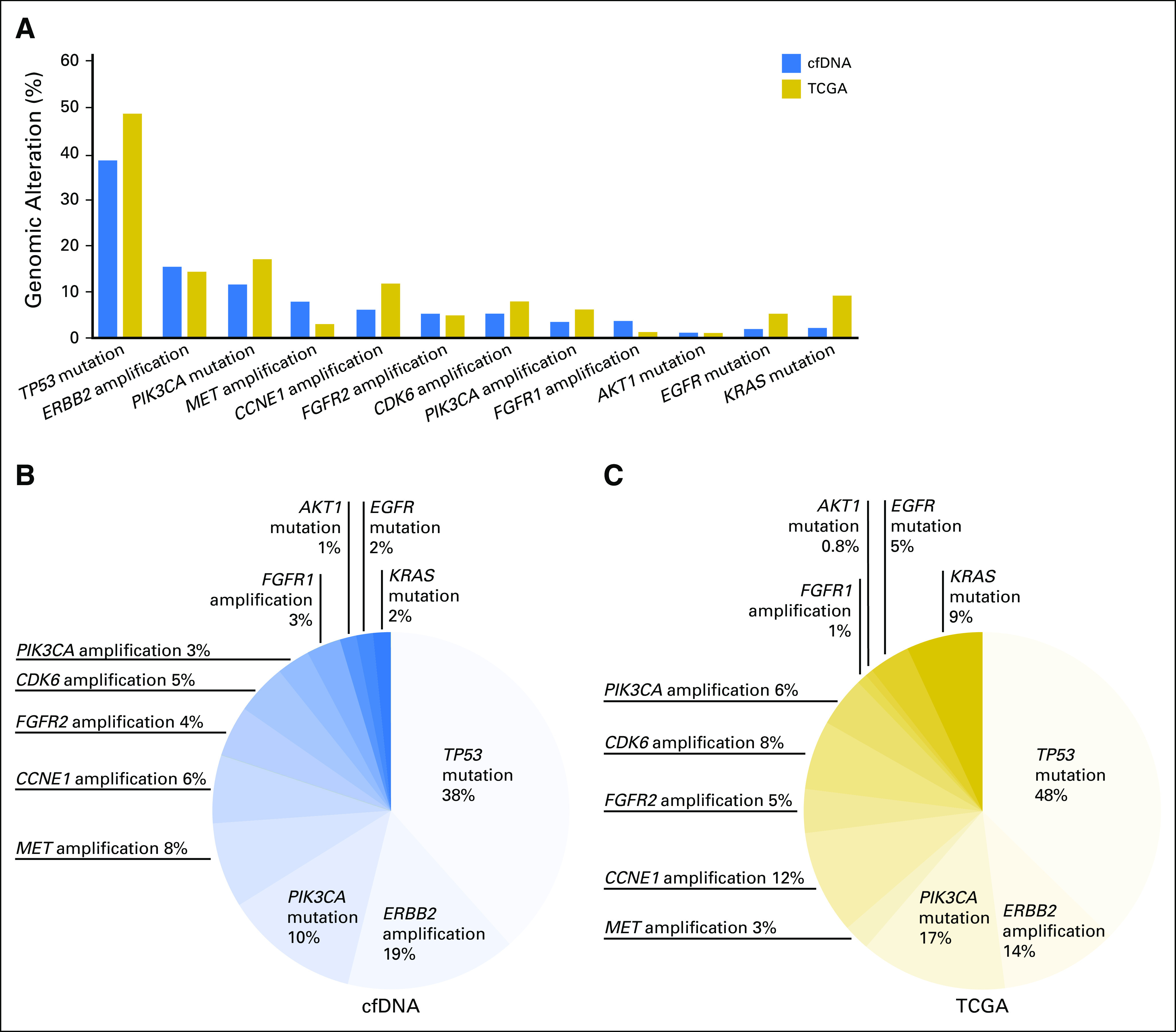
Frequency comparison of cell-free circulating tumor DNA (cfDNA) –detected genomic alterations to The Cancer Genome Atlas (TCGA) in gastric cancer.

ctDNA alterations were detected in 78% of patients with GC (59 of 76 patients), and 33% (25 of 76 patients) had a prespecified GT (Table 2; Appendix Fig A1), as follows: 11 (19%) had *ERBB2* (HER2) CNA (split between at initial diagnosis and at progression [second line or higher]); five (8%) had *MET* CNA (all but one at progression); three (4%) had *FGFR2* CNA (all at progression); and six had point mutations in *PIK3CA* (split between at initial diagnosis and at progression). As shown in Figure 1, the overall distribution of genomic alterations was similar between tumor tissue sequencing results from The Cancer Genome Atlas and ctDNA sequencing in this cohort with GC.

**Table 2.**
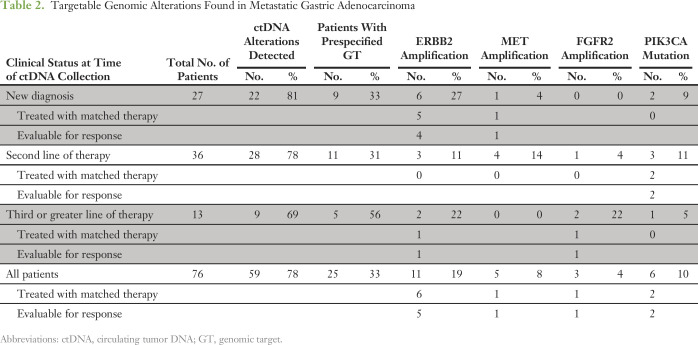
Targetable Genomic Alterations Found in Metastatic Gastric Adenocarcinoma

ctDNA alterations were detected in 85% of patients with NSCLC (62 of 73 patients), with prespecified GTs (Table 3; Appendix Fig A1) in 47% of patients (34 of 73 patients), as follows: 29 patients had canonical *EGFR* driver mutations (exon 19 deletions or SNVs in codons 858, 719, and 861), constituting one third of the newly diagnosed patients and half of the patients evaluated at second line or greater; *EGFR*^T790M^ mutations were found in 17 patients, all at progression; two patients had *EML4-ALK* fusions; one patient had *KIF5B-RET* fusion; and two patients had *ERBB2* insertions (G776 DelinsVC and G778_P780Dup). *ERBB2* (HER2) CNA was identified in two patients at progression (one co-occurring with *EGFR*^T790M^ and one with the *ERBB2* G778_P780Dup), and *MET* was amplified in four patients at progression (one with *ERBB2* insertion and three with *EGFR*^T790M^). However, CNAs in NSCLC were not prespecified GTs.

**Table 3.**
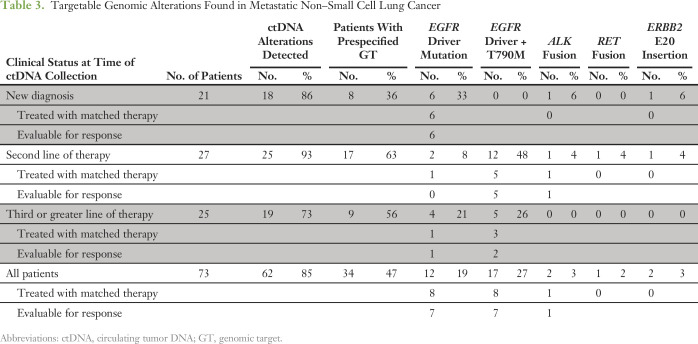
Targetable Genomic Alterations Found in Metastatic Non–Small Cell Lung Cancer

On the basis of rolling substudy availability, inclusion criteria, and patient comorbidities, 10 (40%) of the 25 patients with GC (*ERBB2*, n = 6; *MET*, n = 1; *FGFR2*, n = 1; and *PIK3CA*, n = 2) and 17 (50%) of the 34 patients with NSCLC (*EGFR*, n = 7; *EGFR*^T790M^, n = 7; and *ALK*, n = 1) with prespecified GTs were matched to a molecularly targeted therapy (Tables 2 and 3). Tissue testing was conducted as required by the eligibility criteria for each matched therapy protocol. One patient with GC and two patients with NSCLC were lost to follow-up, leaving nine patients (90%) and 15 patients (88%) evaluable for response, respectively.

### ctDNA-Guidable Targeted Therapies and Response by Cancer Type

**Fig 2.**
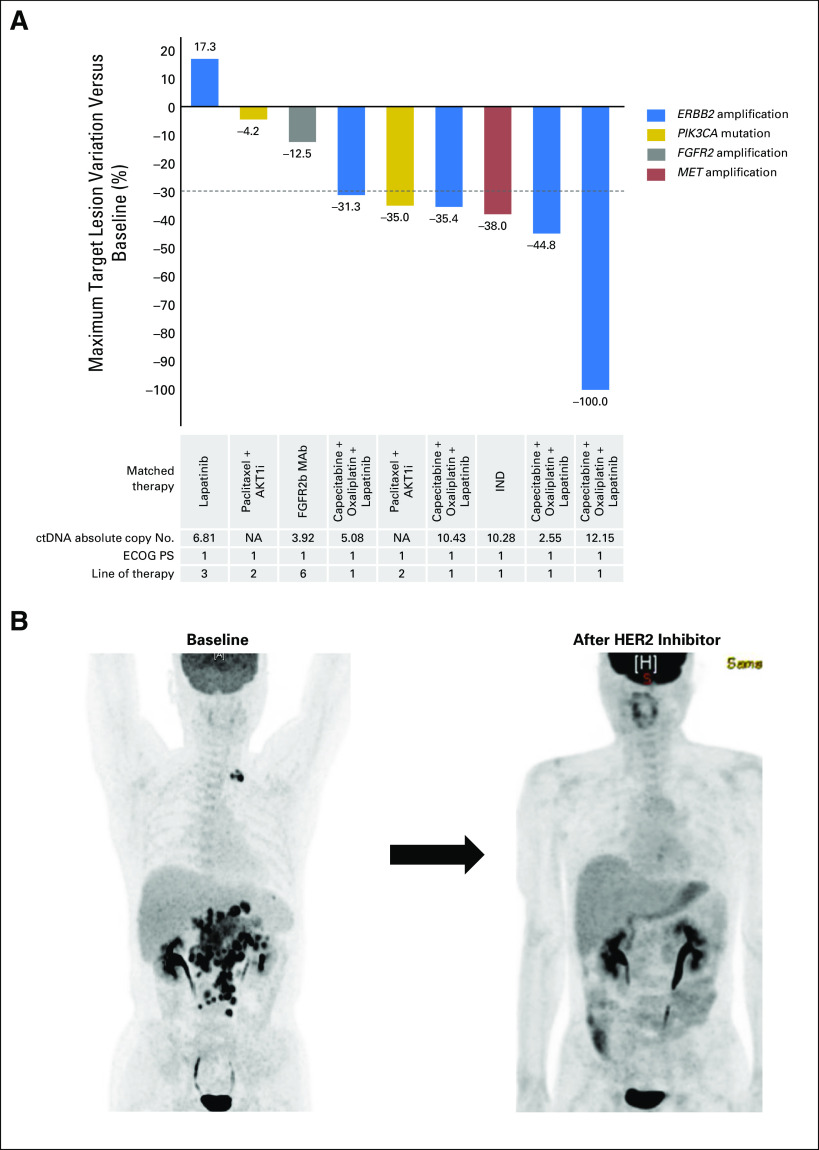

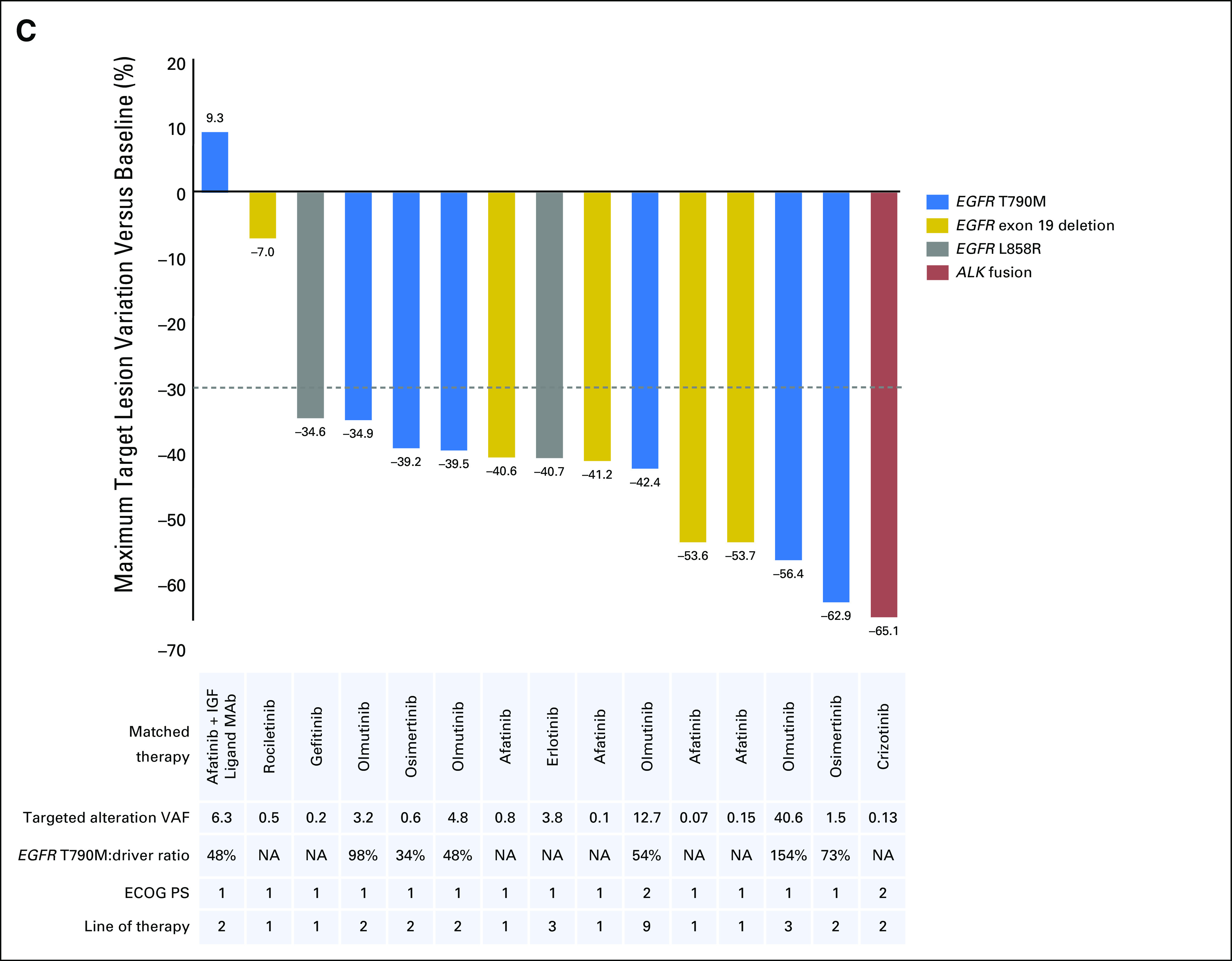
(A) Waterfall plot of response rates in cohort with gastric adenocarcinoma (maximum change in target lesion by alteration). (B) Gastric cancer with complete response targeting *ERBB2* (HER2) gene amplification in plasma. AKT1i, AKT1 inhibitor; cfDNA, cell-free DNA; CNV, copy number variation; CR, complete response; ctDNA, circulating tumor DNA; ECOG PS, Eastern Cooperative Oncology Group performance status; IND, investigational new drug; NA, not applicable; XELOX, capecitabine and oxaliplatin.

In GC, CNAs in *ERBB2* (n = 5), *FGFR2* (n = 1), and *MET* (n = 1) and SNVs in *PIK3CA* (n = 2) were targeted with one patient achieving complete response (CR), five partial response (PR), and three stable disease (SD) for an RR of 67% (95% CI, 31% to 91%) and DCR of 100% (95% CI, 63% to 100%; Table 4, Fig 2A). The absolute copy number in plasma for all focal amplifications was > 4.0 (+++), with two exceptions at 3.92 and 2.55 (++), the former with SD and the latter with PR (Fig 2A). One patient with ctDNA-detected *ERBB2* (HER2) amplification (+++) achieved complete remission after six cycles of capecitabine, oxaliplatin, and lapatinib (Fig 2B).

**Table 4.**
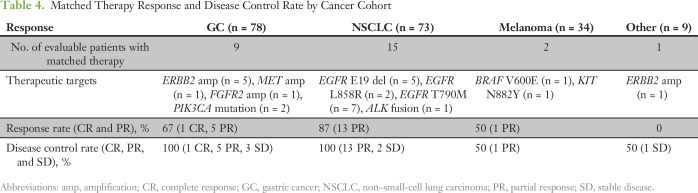
Matched Therapy Response and Disease Control Rate by Cancer Cohort

In NSCLC, *EGFR*^exon19del^ (n = 5), *EGFR*^L858R^ (n = 2), *EGFR*^T790M^ (n = 7), and *ALK* fusion (n = 1) were targeted, with 13 patients achieving PR and two SD for a RR of 87% (95% CI, 58% to 98%) and a DCR of 100% (95% CI, 75% to 100%; Table 4, Fig 2C). The patient with an *ALK* fusion treated with crizotinib achieved a significant 65% response in the target lesion. Of the seven patients receiving first-line epidermal growth factor receptor inhibitors (EGFRi), six achieved PR on afatinib, erlotinib, or gefitinib, whereas the one patient with SD received rociletinib. Similarly, six *EGFR*^T790M^ patients achieving PR were treated with osimertinib or olmutinib, whereas the patient with SD was treated with afatinib plus insulin-like growth factor ligand monoclonal antibody. The targeted alteration VAF ranged from 0.07% to 40.6% ctDNA with no statistically significant correlation between VAF and RECIST response (*P* = .63).

Because of the small treated sample sizes in the other cancer types (two patients with melanoma and one patient with colon cancer), results and discussion of these cases are available in the Data Supplement.

## DISCUSSION

To our knowledge, this is the first prospective ctDNA-guided molecular testing program with objective response evaluated in solid tumors. This program guided patients in whom biopsy was not readily available or in whom tumor material was not sufficient for comprehensive sequencing to genomically matched therapies available in practice or clinical trials. In all, comprehensive ctDNA genomic profiling was feasible, and all samples passed quality control, obviating the need for repeat tests. Of 194 patients, 30 (15.5%) were successfully enrolled onto one of the ongoing matched therapy clinical trials, a rate comparable to tumor sequencing-based trials. Responses to ctDNA-guided matched therapy in GC and NSCLC were similar to those published in tissue-based matched therapy studies, although the sample sizes here are modest.

In GC, CNAs were found in *ERBB2* (HER2), *MET*, and *FGFR2* in 31% of our patients, split evenly between newly diagnosed and pretreated patients, consistent with previous primary tumor estimates of these CNAs at 20% to 22%.^30,31^ Significantly, four (80%) of five patients with *ERBB2* (HER2) -amplified GC responded, including one CR (Fig 2A), with all achieving clinical benefit (CR, PR, or SD). The one patient with GC with *ERBB2* CNA without PR (but with SD) was on lapatinib monotherapy, raising the question of whether chemotherapy produced most of the benefit here. However, addition of lapatinib to chemotherapy did produce a significant overall survival benefit in Asian patients in the Lapatinib Optimization Study in HER2-Positive Gastric Cancer (LOGiC) study.^32^ In addition, a patient with refractory colon cancer with *ERBB2* CNA achieved SD as best response (Table 4). These findings are consistent with the 53% RR recently reported in HER2-positive advanced gastroesophageal adenocarcinoma cancer using capecitabine and oxaliplatin plus lapatinib.^32^ In addition, the 80% RR to targeting ctDNA-detected *ERBB2* amplification in GC here is similar to the RR reported with the same ctDNA test in metastatic breast cancer, where six (86%) of seven patients receiving combination anti-HER2 therapy responded.^25^

The patients with GC with *MET* CNA (+++) and *FGFR2* CNA (++) also achieved clinical benefit with targeted therapy (PR and SD, respectively), although these are not routinely tested for in GC. To enroll patients onto *MET* and *FGFR2* amplification matched trials, we validated these alterations in available corresponding patient tumor tissue. In all, these CNA outcomes add to emerging evidence that high-level ctDNA-detected gene amplifications (++/+++) with this comprehensive digital sequencing method are targetable.^18,25^

In an Asian population with NSCLC, finding *EGFR* driver mutations in 38% of newly diagnosed patients and 50% of patients with progression was expected.^33,34^ All patients with canonical *EGFR* mutations receiving first-line targeted therapy responded except one patient with SD on rociletinib. The partial responses with gefitinib, erlotinib, and afatinib (100%; 95% CI, 52% to 100%) are consistent with the 50% to 70% published RRs with these agents.^35-37^ A single patient with *ALK* fusion achieved good response to crizotinib, as expected for this alteration.^38^ *EGFR*^T790M^ was observed only at progression and was present in 74% of patients with *EGFR* driver mutations (17 of 24 patients) determined at second line or higher, somewhat higher than the 62% rate in the AURA trial.^39^ All patients with *EGFR*^T790M^ mutations (100%; 95% CI, 52% to 100%) had a PR to third-generation EGFRi osimertinib or olmutinib, with one patient stable on afatinib plus a novel insulin-like growth factor-1 ligand monoclonal antibody.

Response to ctDNA-guided matched therapy was independent of the quantitative VAF of the targeted alteration (*P* = .63), as responders had alterations as low as 0.07% (*EGFR*^exon19del^) or 0.13% (*ALK* fusion) and as high as 40.6% (*EGFR*^T790M^). This is consistent with the AURA study findings, in which, with droplet digital PCR hotspot testing, there was no correlation of *EGFR*^T790M^ VAF with response to osimertinib and a patient with VAF as low as 0.03% achieved a response.^23^ Similar-sized tumors may shed variable amounts of DNA into circulation, and ctDNA levels are highly dynamic over time in the same patient, including decreases to low levels in responders.^40,41^

For secondary resistance mutations, the ratio of resistance to initial driver mutation VAF in cfDNA may be a better indicator of response than absolute VAF.^42^ In AURA, a cfDNA ratio > 10% of *EGFR*^T790M^ VAF to *EGFR* driver mutation was a superior predictor of response in plasma *EGFR*^T790M^-positive patients.^23^ All of the patients in our study had ratios of 34% or greater, suggesting that the *EGFR*^T790M^ was relatively clonal and consistent with the high observed RR to third-generation EGFRis (Fig 2C). Thus, an advantage of ctDNA over tissue genotyping is that quantitation of the relative VAFs can provide an indication of the subclonality and potentially predict treatment response, in contrast to a binary positive or negative result. However, a ratio < 10% may be misleading if there is focal amplification of the *EGFR* driver mutation and not *EGFR*^T790M^.^42^

Beyond T790M, recent reports suggest that comprehensive profiling at progression may be important in NSCLC given the multiple other resistance mechanisms after EGFRi therapy.^43^ These include non-*EGFR*^T790M^ on-target point mutations, as well as bypass mutations in *BRAF*, *KRAS*, *MEK*, and *PIK3CA*; CNAs in *MET* and *ERBB2*; fusions in *ALK*; or *RB1* inactivation heralding epithelial to mesenchymal cell transition.^34,44-47^ Because *EGFR*^T790M^ is the resistance mechanism in only half of patients experiencing progression on first-line EGFRi, a comprehensive ctDNA NGS test covering all major types of genomic alterations is particularly relevant.

Small sample sizes for targeted therapy in melanoma and colon cancer limit the conclusions that can be drawn in those cohorts; however, the RR CIs in GC and NSCLC are consistent with tissue-guided matched therapy RRs. All four major alteration types (point mutations, indels, amplifications, and fusions) detected with this comprehensive ctDNA genotyping method had positive responses. Single-arm objective RRs exceeding 30% have led to US Food and Drug Administration regulatory approval of matched therapies.^48,49^ The RRs to ctDNA-detected alterations in this interim analysis (67% [95% CI, 31% to 91%] for GC and 87% [95% CI, 58% to 98%] for NSCLC) support clinical utility for Guardant360 in patients with advanced NSCLC and GC in whom tissue is insufficient or inaccessible and build upon previous validation studies of the diagnostic test used herein.^12,26^

Because this study was not randomized, its primary limitation is the potential for selection bias to enroll patients more likely to benefit. In addition, the cohort is heterogeneous, including patients at varying lines of therapy and with various concomitant treatments, which limits conclusions in this interim analysis. Not all patients with targetable alterations could receive matched therapy because of the various requirements of the multiple parallel matched therapy substudy protocols, performance status, or loss to follow-up. The final analysis will help to address the modest sample size of this interim analysis as well as report on progression-free survival. Future studies should examine ctDNA-guided matched therapy outcomes in more racially diverse cohorts.

To our knowledge, this is the first prospective study to examine the clinical utility of comprehensive ctDNA genomic testing to guide matched therapy selection. The findings here build on cohort studies at other centers demonstrating response to ctDNA-guided matched therapy by the same method in NSCLC and breast cancer.^10,18,25,50^ This study provides additional validation of comprehensive ctDNA genotyping as patients with all four types of genomic alterations had positive responses. ctDNA testing has the potential to reduce biopsies and patient harm,^51^ which is important because invasive biopsies to obtain additional tissue for genotyping are increasing in both clinical practice and research studies.^52,53^Among patients with insufficient tumor tissue for sequencing, ctDNA testing can be a feasible option to guide molecularly matched therapy.
